# Preparative Isolation and Purification of Lignans from *Justicia procumbens* Using High-Speed Counter-Current Chromatography in Stepwise Elution Mode

**DOI:** 10.3390/molecules20047048

**Published:** 2015-04-20

**Authors:** Peijuan Zhou, Qijun Luo, Lijian Ding, Fang Fang, Ye Yuan, Juanjuan Chen, Jinrong Zhang, Haixiao Jin, Shan He

**Affiliations:** 1 Key Laboratory of Applied Marine Biotechnology of Ministry of Education, Ningbo University, Ningbo 315211, China; E-Mails: zhoupeijuanhaha@163.com (P.Z.); 23yuanye@163.com (Y.Y.); jinhaixiao@nbu.edu.cn (H.J.); 2 Laboratory of Marine Natural Products, School of Marine Sciences, Ningbo University, Ningbo 315211, China; E-Mails: 15888107865@163.com (F.F.); chenjuanjuan@nbu.edu.cn (J.C.); zhangjinrong@nbu.edu.cn (J.Z.); 3 College of Pharmacy, Jinan University, Guangzhou 510632, China; E-Mail: huahua20062008@126.com

**Keywords:** *Justicia procumbens*, lignans, high-speed counter-current chromatography, preparative separation, stepwise elution mode

## Abstract

Lignans, which are recognized as main constituents in *Justicia procumbens*, have attracted considerable attention due to their pharmacological activities, including antitumor, anti-hepatitic, cytotoxic, anti-microbial, and anti-virus properties. Preparative high-speed counter-current chromatography (HSCCC) was successfully applied to the isolation and purification of four lignans (justicidin B (**1**), justicidin A (**2**), 6'-hydroxyjusticidin C (**3**) and lignan J_1_ (**4**)) from *J. procumbens* using stepwise elution with a pair of two-phase solvent systems composed of *n*-hexane–ethyl acetate–methanol–water at (1.3:1:1.3:1, v/v) and (2.5:1:2.5:1, v/v). The preparative HSCCC separation was performed on 300 mg of crude sample yielding compounds **1** (19.7 mg), **2** (9.86 mg), **3** (11.26 mg), and **4** (2.54 mg) in a one-step separation, with purities over 95% as determined by HPLC. The structures of these compounds were identified by MS, ^1^H-NMR and ^13^C-NMR. This is the first report on the application of HSCCC to the efficient separation of lignans from *J. procumbens*.

## 1. Introduction

*Justicia procumbens* (Acanthaceae), known as “Juechuang” in Chinese, is one of the most popular traditional Chinese medicines (TCMs) used to treat fever, pain, and cancer [1,2,3]. Its main constituents are lignans and their glycosides [4,5,6,7]. This plant also served as one of the main herbs in *Jian-er syrup*, a Chinese herbal medicine compound preparation [8]. Moreover, many lignans from TCMs are considered lead compounds for the development of new therapeutic agents [9].

In the past few years, the bioactive principles isolated from *J. procumbens* were identified as arylnaphthalide and diarylbutane lignans, and their glycosides [10,11,12]. They exhibit antitumor [13], anti-hepatitis [14], cytotoxic [15], anti-microbial [16], and antiviral activities [17]. This motivated us to develop an efficient preparation method for the bioactive lignans.

**Figure 1:**
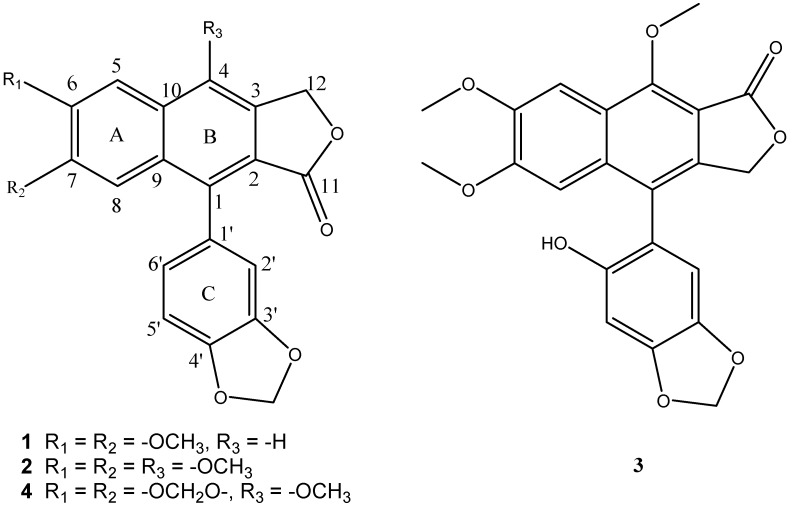
Chemical structures of justicidin B (**1**), justicidin A (**2**), 6'-hydroxyjusticidin C (**3**) and lignan J_1_ (**4**).

Conventional isolation strategies for natural products involve multiple chromatographic steps, which are time consuming and result in substantial loss of samples due to irreversible adsorption [18]. However, high-speed counter-current chromatography (HSCCC), a support-free liquid-liquid partition method, which is free of irreversible adsorption [19], is capable of isolating multiple components from plant extracts with higher recoveries and efficiency [20]. It has been widely used in the preparative separation of various compounds from TCMs and other comprehensive extracts [21,22,23]. The present paper describes the successful preparative separation and purification in one-step of the four lignans justicidin B (**1**), justicidin A (**2**), 6'-hydroxyjusticidin C (**3**) and lignan J_1_ (**4**) (Figure 1) from a crude sample of *J.* *procumbens* using HSCCC in stepwise elution mode for the first time.

## 2. Results and Discussion

### 2.1. HPLC Analysis of the Crude Extract

**Figure 2:**
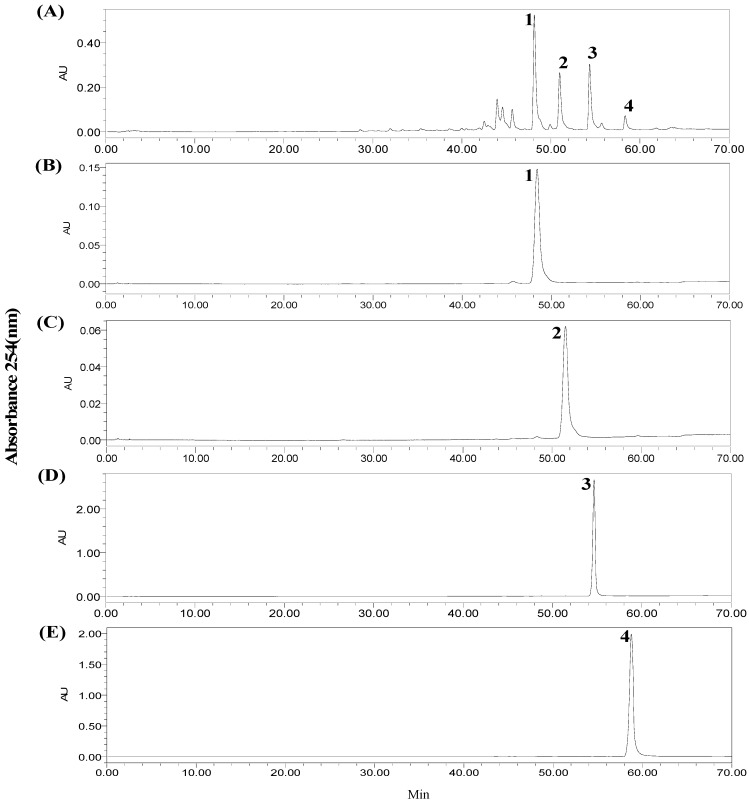
HPLC chromatograms. (**A**) Crude sample from *J.* *procumbens*; (**B**) HSCCC fraction of peak 1 in Figure 3; (**C**) HSCCC fraction of peak 2 in Figure 3; (**D**) HSCCC fraction of peak 3 in Figure 3; (**E**) HSCCC fraction of peak 4 in Figure 3. Conditions: YMC-Pack ODS-A column (150 mm × 4.6 mm I.D., 5 µm); column temperature, 25 °C; mobile phase, methanol and water at the gradient (methanol: 0–60 min, 10%–90%; 60–70 min, 90%); flow rate, 0.8 mL/min; detection, 254 nm.

The crude ethanol extract from *J. procumbens* was first analyzed by HPLC. Different elution modes, flow rates, detection wavelengths and column temperatures were screened. The target components were satisfactorily separated with methanol-water (methanol: 0–60 min, 10%–90%; 60–70 min, 90%) as the solvent system, when the flow rate, column temperature and detection wavelength were set at 0.8 mL/min, 25 °C and 254 nm. The HPLC chromatogram of the crude extract was as shown in Figure 2A. The target compounds were marked as peaks 1, 2, 3 and 4.

### 2.2. Selection of the HSCCC Two-Phase Solvent System

The suitable two-phase solvent system, selected according to the golden rules proposed by Ito, plays a vital role in the HSCCC separation [19]. An ideal two-phase solvent system should satisfy the following requirements: (i) suitable partition coefficient *K*_D_ values of the target compound (usually between 0.5 and 2) [24]; (ii) to separate two compounds, satisfactory separation factor (α = *K*_1_/*K*_2_, where *K*_1_ > *K*_2_) between two components should be greater than 1.5; (iii) relatively short settling time (<20 s) to make sure the retention of stationary phase; (iv) higher retention of the stationary phase provides better peak resolution; (v) sufficient sample solubility; (vi) no sample decomposition [25].

Thus, the preliminary studies were carried out to examine the *K*_D_ values of the lignans **1**, **2**, **3** and **4** in different solvent systems composed of n-hexane-ethyl acetate-methanol-water at various volume ratios by HPLC analysis. *K*_D_ values, expressed as the peak area of the target lignans in the upper phase divided by that in the lower phase were summarized in Table 1.

**Table 1:** *K*_D_ values of the lignans in different volume ratios of the n-hexane-ethyl acetate-methanol-water solvent system for HSCCC separation (compound **1**, justicidin B; compound **2**, justicidin A; compound **3**, 6'-hydroxy justicidin C; compound **4**, lignan J_1_).

*n*-Hexane–Ethyl Acetate–Methanol–Water	*K*_D_						
	Compound 1	Compound 2		Compound 3		Compound 4	
1:1:1:1	>20		>20		>20		>20
1.2:1:1.2:1	1.16		1.89		4.43		13.04
1.3:1:1.3:1	0.93		1.48		3.48		9.79
1.4:1:1.4:1	0.62		1.02		2.35		7.17
1.5:1:1.5:1	0.48		0.76		1.92		4.58
1.8:1:1.8:1	0.19		0.30		0.79		2.34
2:1:2:1	0.12		0.20		0.54		1.49
2.5:1:2.5:1	0.07		0.16		0.31		0.88
3:1:3:1	0.06		0.10		0.28		0.80
4:1:4:1	0.03		0.04		0.12		0.32

As shown in Table 1, none of a suitable two-phase solvent system has *K*_D_ values (compounds **1**–**4)** between 0.5 and 2 at the same time. In other words, the four target compounds could not be separated using a single solvent system [26,27]. To overcome this problem, stepwise HSCCC elution mode was developed, in order to simultaneously separate compounds with largely different *K*_D_ values [28]. This method has been successfully applied to the simultaneous preparative separation of three antioxidative resveratrol oligomers from a wild grape in our previous work [29].

The two-phase solvent system at volume ratio of 1.3:1:1.3:1 or 1.4:1:1.4:1, with the *K*_D_ values of compounds **1** and **2** between 0.5 and 2, were suitable for the separation of **1** and **2**. When the two-phase solvent system 1.3:1:1.3:1 with separation factor α = 1.59 was used, separation of compounds **1** and **2** were achieved with satisfactory resolution and their retention time were shorter than that of the 1.4:1:1.4:1 solvent system. Similarly 2.5:1:2.5:1 were selected for the separation of **3** and **4**.

According to previous literatures and results, we decided to combine the selected solvent systems (1.3:1:1.3:1 and 2.5:1:2.5:1) in one single run. The stepwise elution mode, which provided satisfactory resolution of these four target lignans, involved two steps: the crude sample was first eluted with the solvent system at volume ratio of 1.3:1:1.3:1 until compounds **1** and **2** were eluted out, which was then followed by the mobile phase of the second solvent system with volume ratio of 2.5:1:2.5:1 until compounds **3** and **4** were eluted.

### 2.3. Stepwise HSCCC Separation

**Figure 3:**
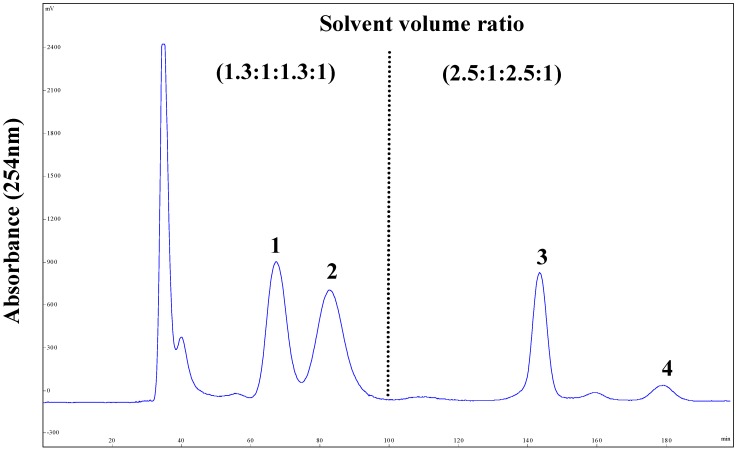
HSCCC chromatogram of the crude extract from *J. procumbens* using stepwise elution with solvent systems A and B. Solvent system A: *n*-hexane-ethyl acetate-methanol-water (1.3:1:1.3:1, v/v/v/v), solvent system B: *n*-hexane-ethyl acetate-methanol-water (2.5:1:2.5:1, v/v/v/v); stationary phase: upper phase of solvent system A; mobile phase: lower aqueous phase of solvent system A and B; column capacity, 320 mL; rotation speed, 1000 rpm; column temperature, 25 °C; flow rate, 3.0 mL/min; detection, 254 nm; sample injected, 300 mg in 5 mL upper phase and 5 mL lower phase; retention of the stationary phase, 56%; separation mode: head-to-tail. Peaks: justicidin B (**1**), justicidin A (**2**), 6'-hydroxyjusticidin C (**3**) and lignan J_1_ (**4**).

Under the optimized stepwise elution mode, the crude sample (300 mg) was dissolved in the 1.3:1:1.3:1 system (10 mL) for the preparative separation by HSCCC with the following condition: rotation speed, 1000 rpm (as recommended by the manufacturer); column temperature, 25 °C; flow rate, 3.0 mL/min; detection, 254 nm. As shown in Figure 3, the separation was started with the solvent system A (1.3:1:1.3:1), and after peaks 1 and 2 were eluted at 68 min and 83 min, the mobile phase was switched (the dotted line in Figure 3) to the lower phase of the solvent system B (2.5:1:2.5:1). Then peaks 3 and 4 were well resolved and eluted in 142 min and 180 min. 

The target compounds **1**–**4** separated by HSCCC were dried under vacuum at 50 °C, redissolved in methanol and detected under the same HPLC conditions for the crude sample. The chromatograms were shown in Figure 2B–E. The results showed that peaks 1–4 were corresponding to the target compounds **1**–**4** in Figure 2. In the stepwise HSCCC separation, the fractions collected from 300 mg crude sample were evaporated to yield compounds **1** (19.7 mg), **2** (9.86 mg), **3** (11.26 mg), and **4** (2.54 mg) with the purities all over 95%. Since the contents of compounds **1**–**4** in the extract were 7.0%, 3.6%, 4.2% and 0.93% respectively as determined by HPLC, their recoveries were 93.8%, 91.3%, 89.4%, and 91.0%, respectively.

## 3. Materials and Methods

### 3.1. Reagents and Materials 

Analytical-grade methanol, ethanol, ethyl acetate, and n-hexane were purchased from Sinopharm Chemical Reagent Co., Ltd (SCRC, Shanghai, China). Reverse osmosis Milli-Q water (18 MΩ) (Millipore, Bedford, MA, USA) was used for all solutions and dilutions. Methanol (chromatographic grade) used for HPLC analyses was purchased from Merck (Darmstadt, Germany). Raw material samples of *J. procumbens* were purchased from Jinhua Pharmaceutical co., LTD. (Jinhua, China.)

### 3.2. Instruments

HSCCC was performed using a model TBE-300C high-speed counter-current chromatography instrument (Tauto Biotech Co. Ltd., Shanghai, China). The apparatus, with a maximum rotational speed of 1000 rpm, was equipped with three polytetrafluoroethylene preparative coils (ID 1.9 mm, total volume is 320 mL) and a 20 mL sample loop. The β values of the multilayer coil varied from 0.5 at internal terminal to 0.8 at the external terminal. Solvents were delivered by a TBP 5002 (Tauto Biotech Co. Ltd) pump. The UV-absorbance of the effluent was measured by a UV 1001 detector (Shanghai Sanotac Scientific Instruments Co. Ltd, Shanghai, China) at 254 nm. A DC-0506 constant-temperature circulating implement (Tauto Biotech Co. Ltd) was used to control the separation temperature. And a N2000 data analysis system (Institute of Automation Engineering, Zhejiang University, Hangzhou, China) was employed for data collection and analysis.

The analytical HPLC system was a Waters Alliance 2695 equipped with a Waters model 2998 diode array detector and Waters Empower System (Waters Co., Milford, CT, USA). ESI-MS analyses were performed using a Thermo TSQ Quantum Access spectrometer with an ESI interface (Thermo Fisher Scientific, San Jose, CA, USA). ^1^H-NMR and ^13^C-NMR spectra were measured on an AVANCE 500 MHz NMR spectrometer with TMS as internal standard at 25 °C (Bruker, Fällanden, Switzerland).

### 3.3. Preparation of Crude Samples

The dried herbs of *J.* *procumbens* were homogenized with a TCM grinder (Yongkang Xi’an Hardware Medical Appliance Factory, Yongkang, China). The powder (1000 g) was extracted with 10 L 95% ethanol twice (each time for 3 h at the room temperature). Then the extract was filtered and concentrated under reduced pressure at 50 °C, yielding a crude sample (30 g) stored at 4 °C for the subsequent HSCCC separation.

### 3.4. Measurement of Partition Coefficients (K_D_)

In order to find a suitable volume ratio (Table 1), the partition coefficients (*K*_D_) of each target compound in different two-phases solvent systems were measured by HPLC as follows: a small amount (1.5 mg) of crude sample was added into a 2.0 mL Eppendorf tube containing 1.5 mL pre-equilibrated two-phase solvent system. After shaking, the mixture was divided into two phases by centrifugation at 3000 rpm for 3 min. Then, an aliquot of each phase (10 μL) was analyzed by HPLC. The *K*_D_ values were defined as the ratio of the peak area of the compound in the upper phase divided by that in the lower phase (Table 1).

### 3.5. Preparation of the Two-Phase Solvent System and Sample Solution 

In this study, the solvent system consisting of *n-*hexane–ethyl acetate–methanol–water (1.3:1:1.3:1, v/v/v/v) was used for the purification of the compounds **1** and **2**. The other solvent system consisting of *n*-hexane–ethyl acetate–methanol–water (2.5:1:2.5:1, v/v/v/v) was used for the purification of the compounds **3** and **4**. The solvent systems were thoroughly mixed, vented in a separation funnel at room temperature, whose upper phase was used as a stationary phase and the lower phase as a mobile phase. Then the two phases were separated and degassed for 10 min before use. The sample solution was prepared by dissolving 300 mg of the crude sample in 10 mL solvent mixture consisting of equal volumes of both upper and lower phases.

### 3.6. HSCCC Separation

In each separation, the coiled column of TBE-300C (320 mL) was entirely filled with the upper phase as stationary phase at a rate of 30 mL/min. Then, the apparatus was rotated at 1000 rpm, while the lower phase (mobile phase) was pumped into the column in a “head to tail” mode at a flow rate of 10 mL/min. After the mobile phase front was emerged and hydrodynamic equilibrium was established in the column, the flow rate was reduced to 3 mL/min. The sample solution (10 mL, 300 mg crude extract) was then injected into the column through the injection valve. All through the experiment, the separation temperature was controlled at 25 °C. The effluent of the column was continuously monitored with a UV detector at 254 nm. Peak fractions were collected manually according to the elution chromatogram. After the separation was completed, the solvents in the column were pushed out [27].

### 3.7. HPLC Analysis and Identification of the Peak Fractions

The peak fractions were evaporated under reduced pressure at 50 °C, and the residues were dissolved in methanol for HPLC analysis. The analyses were performed on a Waters Alliance 2695 liquid chromatography system, equipped with a quaternary solvent delivery system, an autosampler, and a 2998 diode array detector. A YMC-Pack ODS-A column (150 mm × 4.6 mm I.D., 5 µm) at 25 °C was used for all the analyses. Methanol-water was used as the mobile phase in gradient elution mode (methanol: 0–60 min, 10%–90%, 60–70 min, 90%). The flow rate was 0.8 mL/min and the effluent was monitored on-line at 254 nm.

### 3.8. Identification of Target Compounds 

The structural identification of each peak fractions separated by HSCCC was identified according to the analyses of ESI-MS, ^1^H-NMR and ^13^C-NMR data.

Compound **1**. White power, ESI-MS (*m*/*z*): 365.1 (M+H)^+^, 387.1 (M+Na)^+^; The molecular formula was C_21_H_16_O_6_. ^1^H-NMR (CDCl_3_) δ (ppm): 7.72 (1H, s, H-4), 7.20 (1H, s, H-5), 7.12 (1H, s, H-8), 6.98 (1H, d, *J*_6',5'_ = 7.9 Hz, H-5'), 6.87 (1H, s, H-2'), 6.85 (1H, d, *J*_5',6'_ = 7.9 Hz, H-6'), 6.11 (1H, d, *J*_4',3'_ = 1.5 Hz, OCH_2_O-4'), 6.07 (1H, d, *J*_4',3'_ = 1.2 Hz, OCH_2_O-3'), 5.40 (2H, s, H-12), 4.07 (3H, s, OCH_3_-6), 3.83 (3H, s, OCH_3_-7); ^13^C-NMR (CDCl_3_) δ (ppm): 170.0 (C-11), 151.8 (C-7), 150.1 (C-6), 147.6 (C-4'), 147.6 (C-3'), 139.7 (C-1), 139.6 (C-3), 133.2 (C-10), 128.9 (C-9), 128.4 (C-1'), 123.5 (C-6'), 118.5 (C-2), 118.3 (C-4), 110.6 (C-2'), 108.3 (C-8), 106.0 (C-5), 105.9 (C-5'), 101.3 (3'-OCH_2_O-4'), 68.1 (C-12), 56.1 (OCH_3_-6), 55.9 (OCH_3_-7). After comparison with the data given in reference [30], it was identified as justicidin B.

Compound **2**. White power, ESI-MS (*m*/*z*): 395.1 (M+H)^+^, 417.1 (M+Na)^+^; The molecular formula was C_22_H_18_O_7_. ^1^H-NMR (CDCl_3_) δ (ppm): 7.55 (1H, s, H-5), 7.07 (1H, s, H-8), 6.97 (1H, d, *J*_6',5'_ = 7.9 Hz, H-5'), 6.83 (1H, s, H-2'), 6.80 (1H, d, *J*_5',6'_ = 7.8 Hz, H-6'), 6.10 (1H, d, *J*_4',3'_ = 1.5 Hz, OCH_2_O-4'), 6.06 (1H, d, *J*_4',3'_ = 1.5 Hz, OCH_2_O-3'), 5.56 (2H, s, CH_2_-12), 4.14 (3H, s, OCH_3_-4), 4.08 (3H, s, OCH_3_-6), 3.82 (3H, s, OCH_3_-7); ^13^C-NMR (CDCl_3_) δ (ppm): 169.6 (C-11), 151.6 (C-6), 150.3 (C-7), 147.8 (C-4'), 147.5 (C-4), 147.4 (C-3'), 134.4 (C-9), 130.6 (C-3), 128.5 (C-1), 126.0 (C-1'), 124.5 (C-6'), 123.6 (C-10), 119.3 (C-2), 110.8 (C-2'), 108.2 (C-5'), 106.2 (C-8), 101.2 (3'-OCH_2_O-4'), 100.6 (C-5), 66.7 (C-12), 59.7 (OCH_3_-4), 56.2 (OCH_3_-6), 55.9 (OCH_3_-7). After comparison with the data given in reference [5], it was identified as justicidin A.

Compound **3**. Light yellow powder, ESI-MS (*m*/*z*): 433.0 (M+Na)^+^; The molecular formula was C_22_H_18_O_8_. ^1^H-NMR (CDCl_3_) δ (ppm): 7.71 (1H, s, H-5), 6.96–7.02 (2H, m, H-5',2'), 6.82 (1H, s, OH-6'), 6.81 (1H, s, H-8), 6.11 (1H, d, *J*_4'',3'_ = 0.9 Hz, OCH_2_O-4'), 6.08 (1H, d, *J*_4',3''_ = 1.2 Hz, OCH_2_O-3''), 5.15 (2H, s, CH_2_-12), 4.39 (3H, s, OCH_3_-4), 4.07 (3H, s, OCH_3_-6), 3.85 (3H, s, OCH_3_-7); ^13^C-NMR (CDCl_3_) δ (ppm): 169.3 (C-11), 155.5 (C-6), 152.4 (C-7), 149.8 (C-4'), 148.3 (C-4), 147.5 (C-3'), 139.0 (C-9), 133.5 (C-3), 129.7 (C-1), 126.5 (C-1'), 123.7 (C-6'), 123.0 (C-10), 109.8 (C-2), 109.4 (C-2'), 109.0 (C-5'), 104.2 (C-8), 102.3 (3'-OCH_2_O-4'), 101.4 (C-5), 68.9 (C-12), 63.6 (OCH_3_-4), 56.1 (OCH_3_-6), 55.9 (OCH_3_-7). After comparison with the data given in reference [31], it was identified as 6'-hydroxyjusticidin C.

Compound **4**. White powder, ESI-MS (*m*/*z*): 379.0 (M+H)^+^; The molecular formula was C_21_H_14_O_7_. ^1^H-NMR (CDCl_3_) δ (ppm): 7.72 (1H, s, H-5), 7.03 (1H, s, H-8), 6.96 (1H, m, H-6'), 6.76 (1H, d, H-5'), 6.73 (1H, d, H-2'), 6.09 (2H, dd, *J* = 4.6 Hz, *J* = 3.7 Hz, 3'-OCH_2_O-4'), 6.07 (2H, dd, *J* = 4.5 Hz, 6-OCH_2_O-7), 5.12 (2H, s, CH_2_-12), 4.32 (3H, s, OCH_3_-4); ^13^C-NMR (CDCl_3_) δ (ppm): 169.1 (C-11), 150.8 (C-4), 148.2 (C-3'), 147.6 (C-4'), 139.4 (C-2), 138.4 (C-6), 138.3 (C-7), 135.2 (C-9), 129.6 (C-1), 129.4 (C-1'), 127.5 (C-10), 125.4 (C-6'), 122.9 (C-3), 121.7 (C-5), 109.8 (C-2'), 109.0 (C-5'), 101.8 (6-OCH_2_O-7), 101.4 (3'-OCH_2_O-4'), 100.2 (C-8), 68.9 (C-12), 63.6 (OCH_3_-4). After comparison with the data given in reference [32], it was identified as lignan *J*_1_.

## 4. Conclusions

The efficient preparative separation of four lignans from *J. procumbens* by a stepwise HSCCC method has been implemented for the first time. The stepwise elution mode consisted of a pair of two-phase solvent systems composed of *n*-hexane-ethyl acetate-methanol-water at different ratios. The purity of the fractions was over 95% in a one-step separation, demonstrating that stepwise HSCCC is an efficient technique to isolate and purify bioactive compounds with large difference of *K*_D_ values from TCMs. HSCCC could be an important tool for the modernization of TCMs and providing a solution for the sample availability of these pharmacologically important natural products. 
